# Deterministic and Stochastic Cellular Mechanisms Contributing to Carbon Monoxide Induced Ventricular Arrhythmias

**DOI:** 10.3389/fphar.2021.651050

**Published:** 2021-04-28

**Authors:** Moza M. Al-Owais, Derek S. Steele, Arun V. Holden, Alan P. Benson

**Affiliations:** School of Biomedical Sciences, University of Leeds, Leeds, United Kingdom

**Keywords:** carbon monoxide, ion channels, arrhythmias, modeling, potassium channels, action potential duration

## Abstract

Chronic exposure to low levels of Carbon Monoxide is associated with an increased risk of cardiac arrhythmia. Microelectrode recordings from rat and guinea pig single isolated ventricular myocytes exposed to CO releasing molecule CORM-2 and excited at 0.2/s show repolarisation changes that develop over hundreds of seconds: action potential prolongation by delayed repolarisation, EADs, multiple EADs and oscillations around the plateau, leading to irreversible repolarisation failure. The measured direct effects of CO on currents in these cells, and ion channels expressed in mammalian systems showed an increase in prolonged late Na^+^, and a decrease in the maximal T- and L-type Ca^++^. peak and late Na^+^, ultra-rapid delayed, delayed rectifier, and the inward rectifier K^+^ currents. Incorporation of these CO induced changes in maximal currents in ventricular cell models; (Gattoni et al., J. Physiol., 2016, 594, 4193–4224) (rat) and (Luo and Rudy, Circ. Res., 1994, 74, 1071–1096) (guinea-pig) and human endo-, mid-myo- and epi-cardial (O’Hara et al., PLoS Comput. Biol., 2011, 7, e1002061) models, by changes in maximal ionic conductance reproduces these repolarisation abnormalities. Simulations of cell populations with Gaussian distributions of maximal conductance parameters predict a CO induced increase in APD and its variability. Incorporation of these predicted CO induced conductance changes in human ventricular cell electrophysiology into ventricular tissue and wall models give changes in indices for the probability of the initiation of re-entrant arrhythmia.

## Introduction

Carbon monoxide (CO) is a clear, odourless gas, exposure to which presents significant health risks (Sjostrand, 1970). Environmental CO, generated for example, by the incomplete combustion of hydrocarbons found in engine exhaust fumes or by malfunctioning gas appliances, has significant toxicity, with acute CO poisoning accounting for greater than half of all fatal poisonings each year **(** Varon et al., 1999; Reumuth et al., 2019; Kinoshita et al., 2020). As CO demonstrates a far greater affinity for haemoglobin than oxygen, acute exposure can lead to hypoxia (Von Burg, 1999) and an associated increased risk of sudden death (Gandini et al., 2001; Satran et al., 2005). However, the deleterious effects of CO are not just confined to acute exposure at lethal levels. Chronic exposure to CO, at a lower level may cause acute toxicity and can generate neurological and cardiovascular damage (Von Burg, 1999; Gandini et al., 2001; Omaye, 2002; Prockop and Chichkova, 2007) including myocardial injury and fibrosis (Gandini et al., 2001; Henry et al., 2006; Henry et al., 2008). Such sub-lethal CO toxicity has been demonstrated to occur through more complex pathways than tissue hypoxia caused by the displacement of oxygen and increases in carboxyhemoglobin levels (Carnevali et al., 1987; Gandini et al., 2001; Peers and Steele, 2012; Reboul et al., 2012). Exposure to sublethal acute and chronic levels of CO has been shown to often lead to cardiac arrhythmia without correlation to elevated carboxyhemoglobin levels (Carnevali et al., 1987; Gandini et al., 2001; Peers and Steele, 2012; Reboul et al., 2012), particularly where the exposed individual has a pre-existing cardiac condition (Adams et al., 1988; Sheps et al., 1990). Consistently, such exposure manifests with disruption of repolarization and prolongation (accompanied by increased variability or dispersion) of the QT interval (Cosby and Bergeron, 1963; Gandini et al., 2001; Macmillan et al., 2001; Onvlee-Dekker et al., 2007; Sari et al., 2008) Such QT-prolongation has been linked to CO increasing the amplitude of the non-inactivating late Na^+^ current (Dallas et al., 2012), as observed in arrhythmias associated with late Na channel mutations. These effects are linked to the stimulation of local nitric oxide (NO) formation, occurring through CO-mediated activation of neural nitric oxide synthase, resulting in S-nitrosylation of the Na_v_1.5 *α* subunit (Dallas et al., 2012). The proarrhythmic effect of CO through a peroxynitrite-mediated inhibition of *ether-a-go-go related gene* (ERG) K^+^ channel (Kv11.1, KCNH2) contribute significantly in this regard (Al-Owais et al., 2017a).

Interestingly, endogenous CO has also been shown to demonstrate a cardioprotective function (Sjostrand, 1970; Peers and Steele, 2012), conflicting with the deleterious effects frequently encountered from exposure to exogenous CO. Endogenous CO is generated in cardiac myocytes through the activity of the heme-oxygenase enzyme system HO-1 and HO-2 (Tenhunen et al., 1968; Tenhunen et al., 1969; Peers and Steele, 2012). Cardiac insults, such as myocardial ischemia (Lakkisto et al., 2002), have been shown to increase HO-1 levels and overexpression of this enzyme has been demonstrated to limit cardiac ischemia/reperfusion injury-related damage (Clark et al., 2003). In addition, CO exerts a vasodilative effect, increasing cardiac blood flow (McGrath and Smith, 1984; Sylvester and McGowan, 1978). Indeed, it has been demonstrated that CO may also exert cardioprotective action through reactive oxygen species-mediation regulation of the L-type Ca2^+^ channel (Scragg et al., 2008). What remains currently unclear is how discrimination between the beneficial and deleterious effects of endogenous and exogenous CO respectively occurs and what factors are engaged in balancing the conflicting actions on ion channels such as L-type Ca^2+^ (Scragg et al., 2008) and late Na^+^ currents (Dallas et al., 2012) and I_*kr*_ (Al-Owais et al., 2017a) in cardiac myocytes. In a non-smoker healthy adult the normal ranges for CO in the blood measured using carboxyhemoglobin (COHb) level is less than 2.3%, and in adult smokers is between 3–5 and 5.6% in those who smoked one pack per day (Adams et al., 1988) higher levels may indicate CO intoxication/poisoning. *In vitro* experiments, summarized in Table 1, indicated that increased CO has changed the maximum rates of depolarization and repolarization, decreased the peak to peak amplitude of the action potential, and prolonged action potential duration (APD) in these tested cell types, in spite of their different ionic current profile and shape of APs (Varró et al., 1993; Rosati at al., 2008); The APD prolongation is large enough to mimic pathological changes, the % of change increase in APD are in the ranges seen in congenital and drug induced LQT syndromes, and so may be proarrhythmogenic. CO poisoning has been described as mimicking long-QT (LQT) syndrome (Onvlee-Dekker et al., 2007). A simple explanation for these effects is that exposure to low levels of CO alters the magnitude or kinetics of membrane ionic currents. The actions of CO on ionic currents have been characterized by whole cell and patch voltage clamp experiments as listed in Table 2.

**TABLE 1:** summary *in vitro* changes in action potential characteristics produced by CO.

Cell type/Conditions	Rat ventricular myocytes	GP ventricular myocytes	HL-1 cardiac muscle cell line
Parameter	Room temperature	Room temperature	Room temperature
	Evoked APs every 2 s, CORM-2 (10–30 μM)	Evoked APs every 6 s CORM-2 (10 µM)	Spontaneous activity CORM-2 (30 µM)
**APD**_**50**_	↑38 ± 3% (*p* < 0.01; *N* = 6)	↑57.5 ± 15.8 (*p* < 0.001; *N* = 14)	↑22.3 ± 2.6% (*p* < 0.001; *N* = 7)
**APD**_**90**_	↑50 ± 6.5% (*p* < 0.01; *N* = 6)	↑59.4 ± 15.1 (*p* < 0.01; *N* = 14)	↑18.6 ± 6.8% (*p* < 0.01; *N* = 7)
	↑68.7 ± 4.54% (*p* < 0.001; *N* = 5)		
**AP amplitudes**	↓17.7% (*p* < 0.05; *N* = 20)	↓12.56% (*p* < 0.01; *N* = 14)	↑ 32 ± 8% (*p* < 0.01; *N* = 7)
**Ref**	Dallas et al. (2012); Liang et al. (2014)	Al-Owais et al. (2017a)	Al-Owais et al. (2017b)

**TABLE 2:** summary changes in maximal ionic currents from literature induced via CO.

Gene/Subunit protein	Ion channel	CO effect ↓ (inhibition) ↑ (increase)	Type of cell tested	CORM-2 IC50	Ref
CACNA1C/Cav1.2	L-type Ca channel	↓53.2 ± 2.8% (30 μM CORM-2; *N* = 10; 5 mM Ca)	Rat Myocytes	14.8 ± 0.9 µM	Scragg et al. 2008
		↓44.5 ± 8.3% (CO saturated Solution; *N* = 6; 2 mM Ca)	Rat Myocytes	not measured	Uemura et al. (2005)
		↓12.5% (3 µM CORM-2; *N* = 4; 1.8 mM Ca)	Rat Myocytes	not measured	This study_Supplementary Figure S2
SCN5A/Nav1.5INalate	Fast Na responsible for the rapid depolarization (phase 0)	↓53.4 ± 7.7% (30 μM CORM-2; *N* = 6)	Rat Myocytes	not measured	Dallas et al. (2012)
	Non inactivating Late component	↑105.7 ± 31.15 (30 μM CORM-2; *N* = 5)			
KCNH2/Kv11.1/Ikr	hERG channel mediates the repolarizing IKr currents	↓65.22 ± 4% (*N* = 6; 3 μM)	HEK293	1.6 μM	Al-Owais et al. (2017a)
		↓43.5 ± 2.3% (*N* = 11; 10 μM)	Guinea pig myocytes	not measured	
CACNA1H/Cav3.2	T-type calcium current	↓ 47.5 ± 3.5% by CORM-2 (*N* = 5; 3 μM)	HEK293	3 μM	Boycott et al. (2013)
KCNJ/Kir2	mediates Ik1 Inward-rectifier potassium ion channel	↓34.43 ± 4.27% (10 μM CORM-2; *N* = 5)	Rat Myocytes	not measured	Liang et al. (2014)
		↓35.6 ± 3.24% (1 μM CORM-2; *N* = 5)			

In this study we aim to use the CO induced changes in maximal ionic currents obtained from voltage clamp experiments on isolated myocytes, summarized in Table 2, to predict a possible anti- or pro-arrhythmogenic change in human cell and tissue action potentials. We incorporate these CO induced changes as changes in maximal conductance in computational models for rat, guinea pig and human ventricular cells, and examine evoked action potentials and their properties. The objectives are to see if these changes can reproduce the CO effects on action potential morphology seen *in vitro* in rat and guinea pig ventricular myocytes summarized in Table 1; to compute the effects of rate and variability in membrane conductance parameters; and examine the dynamics of action potential prolongation, early afterdepolarizations (EADs) and plateau oscillations produced by the effects of CO; to compute the effects of endogenous CO on human cell and propagating action potentials and to consider if these changes in tissue properties would be proarrhythmogenic.

Although spontaneous cardiac arrhythmia are a common cause of premature death, and account for 15–20% of premature deaths in the adult population, these deaths are rare events, occur in a year at about 50 per 100,000 adults, and in an individual once in about 10^9^ heartbeats. Clinical recordings of the ECG during a spontaneous arrhythmia ending in death have been captured in hospital, during Holter recordings, and by implanted pacemaker/defibrillator/resynchronization therapy devices, however, they are rare and only allow descriptive and correlational studies.

Clinical and animal observational studies of arrhythmia induced by high frequency pacing of the myocardium are common, and experiments on the termination (defibrillation) of induced arrhythmia can be carried out *in vivo* (Holden et al., 2019), in isolated, perfused hearts (Nanthakumar et al., 2007), and in computational models of the ventricles (Trayanova et al., 2017). Arrhythmia can be induced by appropriately timed single premature or rapid electrical stimulation pacing during *in vivo* or *in vitro* experiments, and pharmacological effects on their initiation threshold and persistence characteristics quantified. The mechanisms and development of spontaneous cardiac arrhythmia can be studied by computational modelling, of myocardial cells and tissues, informed by the applied mathematics of nonlinear dynamics, nonlinear waves (Cherry et al., 2012) and stochastic processes (Colman et al., 2021).

## Methods

### Myocytes Isolations and Electrophysiologic Techniques

Ventricular myocytes were isolated as described previously (Dallas et al., 2012). Animals were humanely euthanized in accordance with United Kingdom Home Office Guidance on the Operation of Animals (Scientific Procedures) Act 1986 and with university of Leeds Ethical Review Committee approval (AWCNRWDS130706). Human induced pluripotent stem cell-derived cardiomyocytes (Sigma-Aldrich, United Kingdom) were cultured as described previously (Stevens et al., 2009; Emre et al., 2010) on precoated coverslips with matrigel (BD Biosciences, United Kingdom) in RPMI media (Gibco, United Kingdom) supplemented with B27 (Thermo Fisher Scientific, United Kingdom) and ROCK inhibitor (Sigma-Aldrich, United Kingdom). After 24 h media was replaced with fresh RPMI-B27 media and cells were grown further, with media being replaced every 48–72 h. Some experimental data presented here were obtained from earlier projects (Dallas et al., 2012, Al-Owais at al., 2017a; Yang et al., 2017) which had received prior ethical approval from the Faculties of Biology, Medicine and Health Sciences, and University of Leeds Ethical committees. Whole cell patch clamp recordings were made at room temperature (21–23°C) under voltage or current clamp using patch pipettes with 2–4 MΩ resistance. For L-type Ca^2+^ current whole cell recording, cells were perfused in extracellular solution (NaCl 140, CsCl 5.4, CaCl_2_ 1.8, MgCl_2_ 1, HEPES 10, and glucose 10; in mM, pH 7.4) and intracellular solution (CsCl 115, HEPES 10, EGTA 10, tetraethylammonium chloride 20, MgATP 5, CaCl_2_ 1; in mM, pH 7.0) was used to fill patch pipettes, cells were voltage-clamped as described in Scragg et al. (2008). Action potential were triggered by 5 ms current pulse in current clamp mode., Patch electrodes were filled with (mM): 10 NaCl, 130 KCI, 5 EGTA, 5 HEPES, 5 MgATP, 1 CaCl_2_, 1 MgCl_2_; pH 7.2. The extracellular solution contained (mM): 120 NaCl, 5KCI, 1.8 CaCl_2_, 1 MgCl_2_, 10 HEPES, 10 glucose; pH 7.4. Stocks of carbon monoxide releasing molecule CORM-2 were made up fresh as 30 mM stocks and used for the duration of the experiment via application through the external solutions at desired concentrations (3 µM for currents recording or 10 µM for action potentials recording). Signals were acquired using Axopatch 200B controlled by Clampex 10.0 software and a Digidata 1322A interface (Axon Instruments, Inc., Foster City, CA). Clampfit 10 (Axon Instruments) was used for an offline analysis. *In vitro* experimental data are expressed as mean ± SD, and the number of cells ‘N’ or action potentials ‘n’, is in the figures or tables. Statistical analysis was performed using Student’s t-tests, where *p* < 0.05 was considered statistically significant.

### Computational Modelling

The control behavior of single ventricular myocytes were modelled by rat: the Gattoni et al. (2016) model, with standard parameter values, guinea pig: the Luo and Rudy (1994) endocardial, midmyocardial and epicardial cell dynamic models, with standard parameters from http://rudylab.wustl.edu/research/cell/methodology/cellmodels/LRd/code.htm and human: the O’Hara et al. (2011) endocardial, midmyocardial and epicardial cell models, with standard parameters. These models are all parameterized for 36**°**C. The effects of prolonged exposure to CO were incorporated by scaling the maximal conductance G_Na_ by −54%, G_Nal_ by +106%, G_CaL_ by −53%, G_kr_ by −44%, and G_k1_ by −34%. The effects of NS1643 on channel kinetics were modeled as in Peitersen et al., 2008. Propagation was modelled by the one-dimensional non-linear partial differential equation:

$$\partial V/\partial t\;=\;\nabla \left(D\nabla V\right)-I_{ion}$$ (1)

*V*/mV is membrane potential, ∇ is a spatial gradient operator, and *t* is time/ms. *D* is the diffusion coefficient/mm^2^ms^−1^ that characterizes the electrotonic spread of voltage. *I*_ion_/μA.μF^−1^is the total membrane ionic current density. In the human ventricular wall model the diffusion coefficient D = 0.048 mm^2^/ms as described in Colman et al. (2017). There was a stepwise change in the cell parameters between the endocardial, midmyocardial and epicardial models, each of which occupied a third of the 18 mm 1-D strand. The vulnerable window was computed as in Holden et al. (2006). Cell models and tissue models were solved with a space step of Δx = 0.2 mm, an adaptive time step of 0.01–0.25 ms. Cell model conductance parameters were Gaussian distributed with a ±5% standard deviation.

The data in Tables 1, 2 are from experiments in which action potentials were excited and voltage clamp pulses were applied periodically, at cycle lengths of 2–6 s, to allow full recovery between pulses. To explore possible proarrhythmogenic processes the cell models were excited periodically at cycle lengths that decreased from 6 s to 200–300 ms, until the model failed to produce action potentials after every stimulus. The restitution curves cover the low rate experimental range and the higher rate physiological range of *in vivo* cycle lengths in rat, guinea pig and human. The modelling does not include any adrenergic direct effects on ventricular cell electrophysiology, that would be present at high heart rates *in vivo*.

## Results

### Carbon Monoxide Prolong Action Potential Duration in Rat Ventricular Myocytes

**FIGURE 1:**
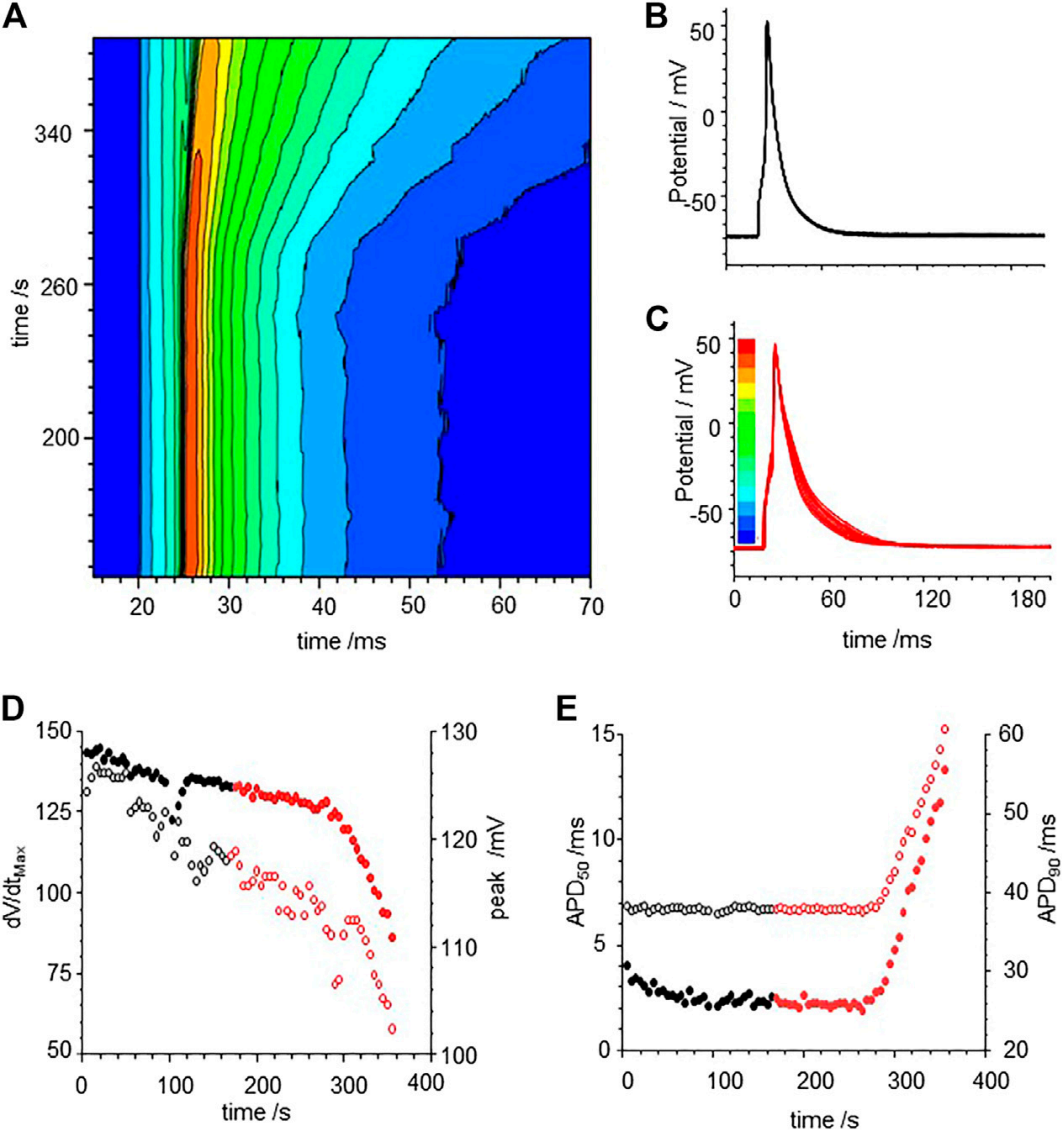
Carbon monoxide (CO) extended action potential durations (APD) in rat ventricular myocytes. **(A)** color coded mapping of smooth prolongation of action potentials during 200 s of triggered activity, preceding and during perfusion of CO releasing molecule CORM-2 (10 µM). Action potential variability before **(B)** and after **(C)** exposure to CORM-2: superimposed action potentials during initial 100 s, cell was paced once every 5 s with CORM-2 added to perfusate at time 20 s, after 170 s of pacing, **(D)** Time series plot of peak depolarization (filled symbols) and dV/dt_max_ (open symbols). **(E)** Time series plot showing APD_50_ (open symbols and APD_90_ (filled symbols) before (black symbols) and during (red symbols) exposure to CORM-2.

Exposure to CO via application of CO releasing molecules CORM-2 produces a smooth, slow, two-fold prolongation in the evoked action potential durations measured at 50% (APD_50_) and 90% (APD_90_) of maximal amplitude, as illustrated in Figure 1. During the first 250 s of the experiment, illustrated in Figure 1, before the CORM-2 was added, the APD_90_ decreases by 15% and can be fitted by a single exponential with a time constant of 37.7 ± 5.3 s, and by 50 s the cell has equilibrated. The APD_90_ time series, from 50 to 250 s, is stationary, with a coefficient of variation of 2% and a mean APD_90_ of 28.1 ms. Following CO perfusion via CORM-2, the APDs lengthen progressively, doubling over 50 s, (Figure 1A). dV/dt_max_ decreases linearly with time, from 110 to 50 V/s, the peak-to-peak amplitude decreases by 7 mV and the latency increases by 2.3 ms over 90 s (Figure 1A,B). The decrease in amplitude of the action potential is due to a decrease in its peak value. The peak of the action potential and dV/dt_max_ change with the same time course, which may be interpreted as the time course of a change in maximal G_Na_. Such a decrease in maximal G_Na_ would also explain the increase in latency. The response to CO of the same ventricular rat myocyte example is quantified in Figures 1D–F. In all experiments before CORM-2 was applied the APD_50_ and APD_90,_ fluctuated, with coefficients of variation of < 5%. These fluctuations are stationary in the mean and standard deviation and may reflect intrinsic stochasticity in the electrochemical activities (membrane currents and intracellular ionic activities) of the isolated cell under periodic forcing at a constant rate. The stationarity of the APDs for over 200 s, before CORM-2 was applied, suggests that the slow increase in APDs seen after switching was a response to the CO perfusion and not the result of the prolonged period of pacing (Figures 1B,C).

**FIGURE 2:**
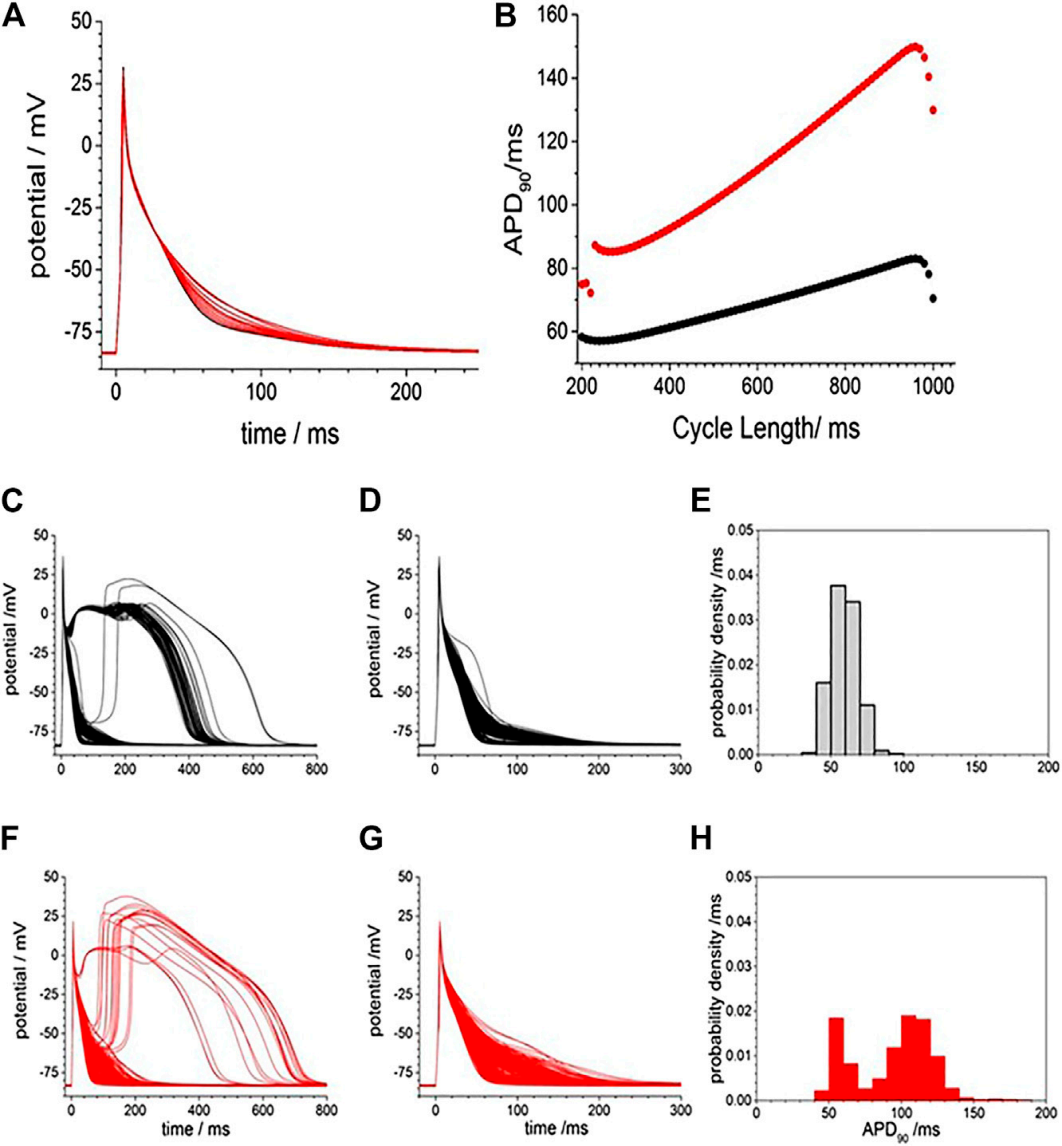
Gattoni et al. model generated potentials increased in duration and variability with CO. **(A)**, smooth prolongation of action potential produced by scaling, by 0–100%, of the CO conductance effects. Periodic action potentials, BCL 1000 ms, after 100 cycles of pacing; **(B)** Dynamic APD_90_ restitution curve showing the effects of CO (red) when compared with standard parameters (black). APD variability with standard model (black) and with CO parameter changes (red) and Gaussian distributed mean parameters with ±5% variability in all model conductance parameters **(C)**. 500 action potentials produced after 50 cycles with BCL 950 ms **(D)**. Action potentials and **(E)** probability density estimate for APD_90_ after atypical action potentials with APD_50_ > 100 ms excluded **(F–H)** as in **(C–E)** with mean parameters for CO.

To examine if a similar effect can be produced *in silico*, we included membrane currents conductance change by CO observed by us and others (Table 2) into a rat ventricular cell models (Gattoni et al*.,* 2016). The conductance changes produced by CO prolong the APD at all BCLs between 200 and 1000 ms (Figure 2A), but not by the two-fold increase seen in experiments. The increase in APD_90_ is seen throughout the dynamic restitution curve, where at BCLs of 300 and 900 ms the APD_90_ it increased by 41 and 65%. The action potential shape, and the APD_90_ variability in a population of Gattoni et al., 2016 cell models with current density parameters (G_Na_, G_NaL_, Gto. PCaL, GKr, GKs, G_K1_, G_NaCa_, G_NaK_), background currents (G_Nab_, G_Cab_.G_Kb_), sarcoplasmic Ca pump, and calcium handling parameters drawn from a Gaussian distribution with 5% variability about their standard values was greater in the cell population with CO parameters than in the population with standard parameters., (Figures 2C–G). Atypical triggered responses, with a guinea pig AP-like plateau, or with a rat-like AP followed by a DAD-like event, starting 100–200 ms after the initial upswing of the AP, and lasting 400–800 ms, were occasionally produced in both the standard population and CO populations (Figures 2C,F). After these were excluded (all APD_90_ > 200 ms excluded), the population of cell models with CO parameters had APD_90_ (93.5 ± 27.5 ms, *n* = 477) almost double that of the population with standard parameter (59.2 ± 9.2 ms; *n* = 433; Figure 2D–G).

### Carbon Monoxide Prolongs Action Potential Duration and Induced Early After-Depolarizations in Guinea Pig Ventricular Myocytes

**FIGURE 3:**
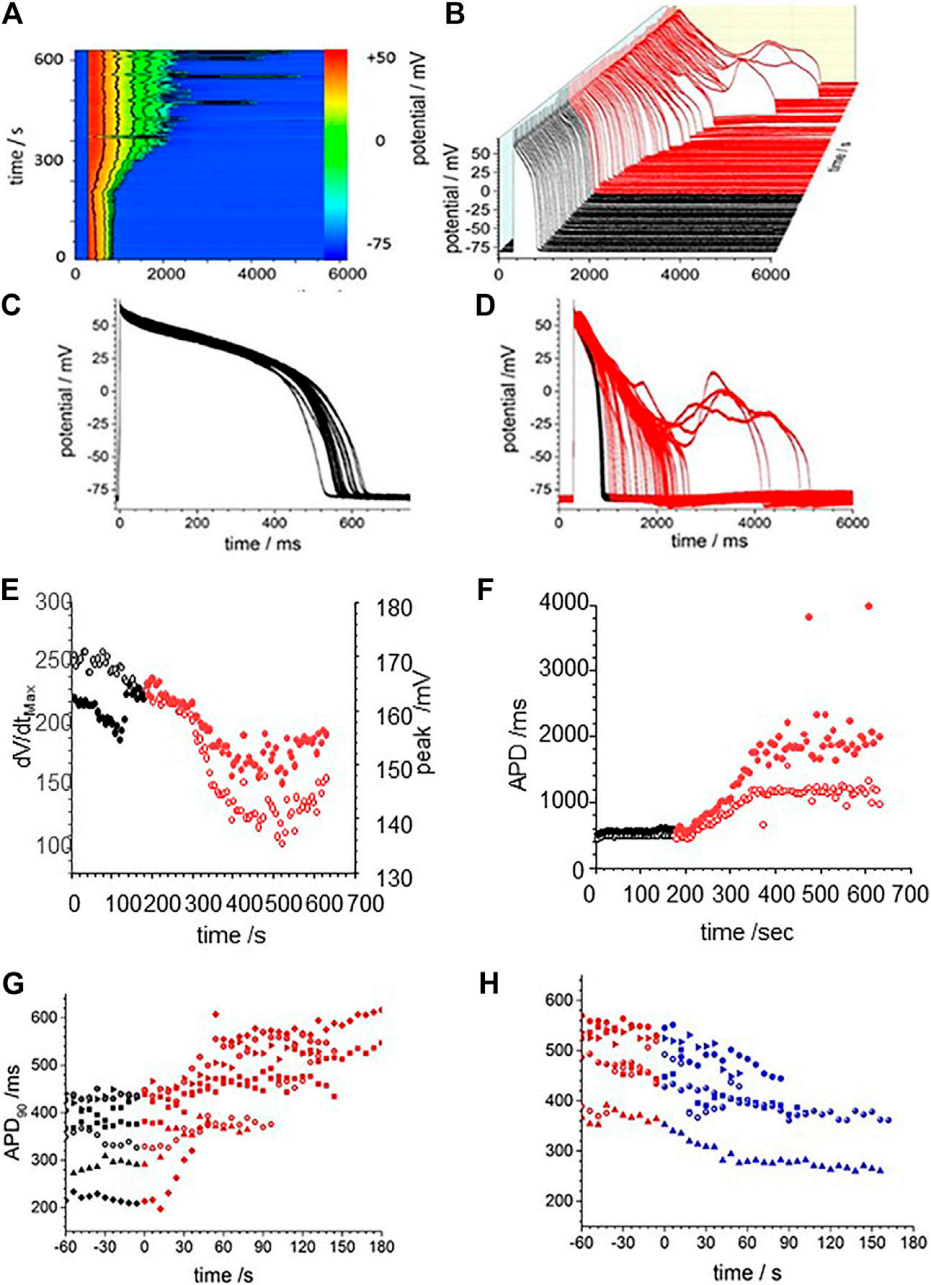
CO prolongs action potentials of isolated guinea-pig ventricular myocyte. **(A)** Color map of membrane potential during 105 cycles of pacing, with BCL of 6 s **(B)** staggered plot of V(t) during these action potentials. Black before effect of CO, red during development of effect of CO. CORM2 was perfused into the bath supply line at time 180 s **(C)** Intrinsic fluctuations in APD_90_ before effect of CO. **(D)** Superposition of evoked actions potentials before and during CORM2. Time series plot of **(E)** peak depolarization (filled symbols) and dV/dt_max_ (open symbols) measurements and **(F)**; APD_50_ (open symbols and APD_90_ (filled symbols) before (black symbols) and during (red symbols) the response to perfusion by CORM-2. **(G)** APD_90_ during application of CORM-2 and **(H)** NS1643, starting with last 10 action potentials before perfusion with CORM-2, and during perfusion with NS1643.

Figure 3 illustrates the action potential responses of isolated GP ventricular myocyte to CORM-2. Before exposure to CORM-2 the action potentials triggered by each stimulus are variable, with a stationary APD_90_ sequence (Figure 3A–C). The intrinsic variability of the APD_90_ of the cell before exposure to CORM-2 illustrated in Figure 3, a-f had a coefficient of variation of 2.5% (487.12, ±11.6, *n* = 30). When CO was applied via CORM-2, a gradual progressive, lengthening of the APD_90_ of the myocytes was produced, almost doubling the duration; from 484.2 to 768.5 ms in 236 s in the example shown (Figure 3A–D), and from 419 ± 44 ms to 708 ± 125 ms (*n* = 14, *p* < 0.01), over 200–300 s in all the cells where CORM-2 perfusion was maintained until EADs occurred. The progressive lengthening the APD_90_ (Figure 3F) was mirrored by a decrease in dV/dt_max_, from 245.2 ± 11 to 160.8 ± 38.8 Vs^−1^ (Figure 3E). After several 100 s of exposure to CO occasional EADs were observed in all cells, with the duration of the triggered response increasing up to 5 s (see Supplementary Figure S1). An experiment was stopped when there was failure to repolarize before the next stimulus. There could be multiple EADs/oscillations in one triggered response, and responses containing EADs or oscillations occurred intermittently with time. The periods between the peaks of any two EADs in one cell, and these periods in subsequent evoked responses, were not the same, but were all around 1–2 s, the peak of action potentials was reduced by ∼10 mV and APD_90_, even excluding EADs, was more than doubled (Figures 3E,F). In experiments where a shorter exposure to CORM-2 (< 200 s) did not allow EADs to develop the ERG activator, NS1643 partially or completely reversed the APD_90_ increases (Figures 3G,H).

**FIGURE 4:**
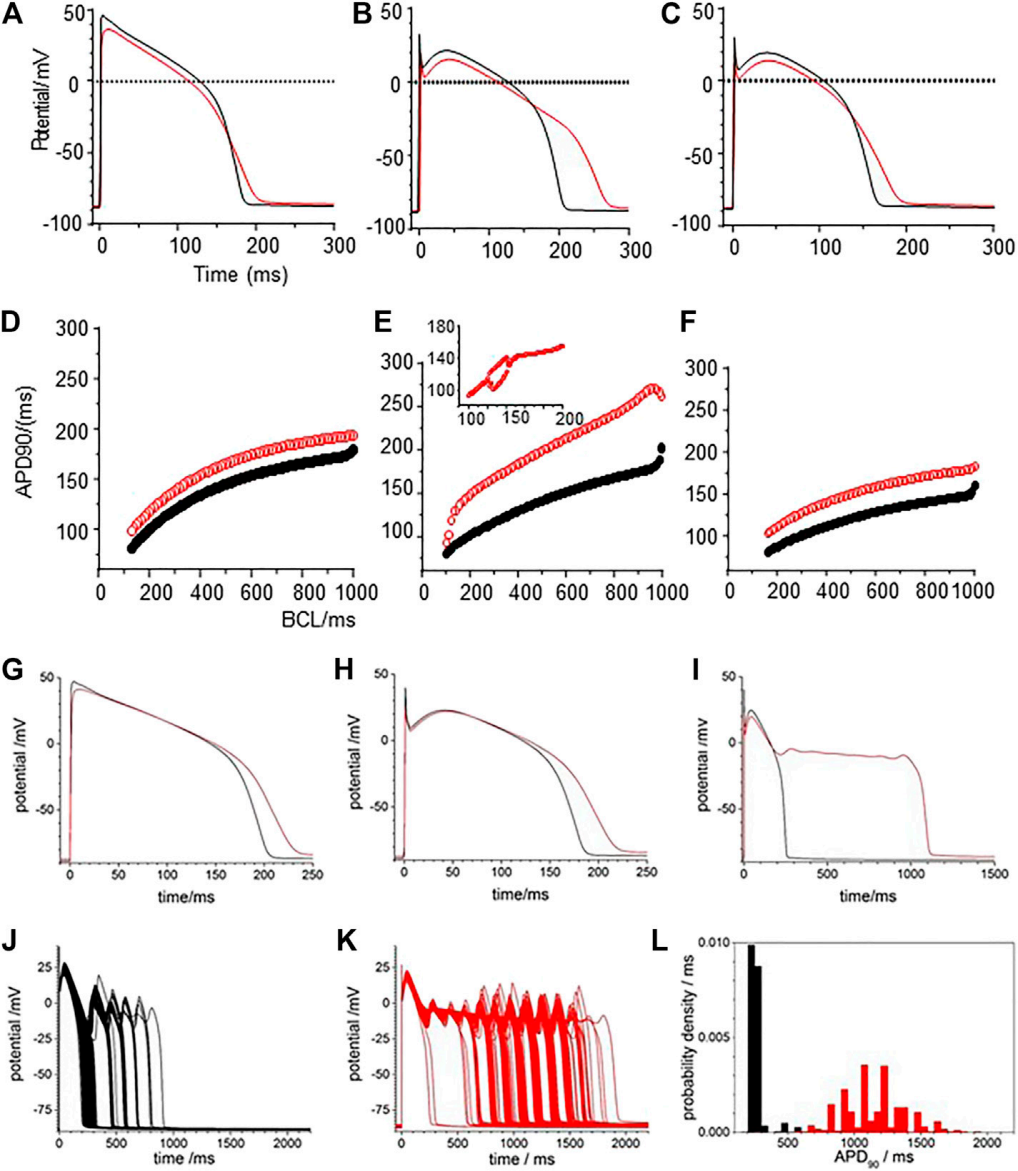
CO prolongs APD90 in LRd (1994) ventricular cell models. Periodic action potentials at BCL of 1 s for **(A)** endo-, **(B)** midmyo **(C)** epi-myocardial cell models 100th action potential at BCL of 1 s and **(D–F)** dynamic restitution curves for endo-. midmyo- and epi-cardial cell models. Oscillatory EADs develop in mid-myocardial, but not epi- and endo-, cell models a BCL of 6 s 100th triggered action potentials at BCL of 6 s for **(G)** endo-, **(H)** epi- and **(I)** mid-myocardial cell models. 1,000 superimposed action potentials, each the 100th action potential at a BCL of 6 s, for standard LRd midmyocardial model with all the current magnitude parameters Gaussian distributed with a 5% standard deviation about **(J)** its standard values **(K)** modified for the effects of CO. **(I)** Probability density histograms for APD_90_ of midmyocardial action potentials. Red: with CO parameters, black standard parameters.

The effects of CO were modelled by the changes in maximal G_Na_, G_CaL_, G_kr_, and G_k1_ conductance changes in Table 2: there is no explicit representation of G_Na,l_ in the LRd model. Figure 4 presents numerical solutions of the effects of CO in the LRd model, the action potentials for all three cell sub-types are prolonged by CO, and the plateau potential depressed, with the largest increase in the APD_90_ observed in the M-cell model (Figures 4A–C). The increased APD_90_ is also seen in the restitution curves at all BCL (Figures 4D–F). The restitution curve of the M cell with CO parameters shows alternans at short cycle lengths of 100–200 ms. The APD_90_ of both standard and modified for CO cell models, computed for a body temperature of 38**°**C, are much less than the observed APD_90_ of Figure 3, recorded at room temperature (∼20**°**C), and no EADs were observed in the dynamic restitution curves of Figure 4D–F.

The BCL in the *in vitro* experiments was 6 s, to allow full recovery after each triggered action potential, evidenced by the stationarity of the control action potentials and APD_90_ time series of Figure 3. Pacing the LRd models with a BCL of 6 s gave a greater increase of the APD_90_ by CO, and a greatly extended plateau in the M cell model (Figure 4G–I).

Figure 4J–L represent the activity of populations of midmyocardial cells paced with a BCL of 6 s, with conductance parameters Gaussian distributed about, the standard or CO modified value and a 5% standard deviation. Both the standard and CO cell populations exhibit characteristic action potentials with a fast depolarization, slow repolarization to ∼−20 mV, and faster repolarization to the resting potential, and action potentials with EADs, multiple EADs and oscillatory plateau qualitatively similar to those seen in Figure 3 and Supplementary Figure S1. Histograms of the APD_90_ for the standard and CO cell populations are unimodal and positively skewed, and the APD_90_’s of the CO population have a five-fold longer mean (1,164.5 ± 230.6 compared to 205.9 ± 18.0 ms, *n* = 1,000) and a broader, multimodal distribution. The effect of the CO is to increase the APD at all rates in the cell models, and to increases the variability in a population of models (Figure 4L).

**FIGURE 5:**
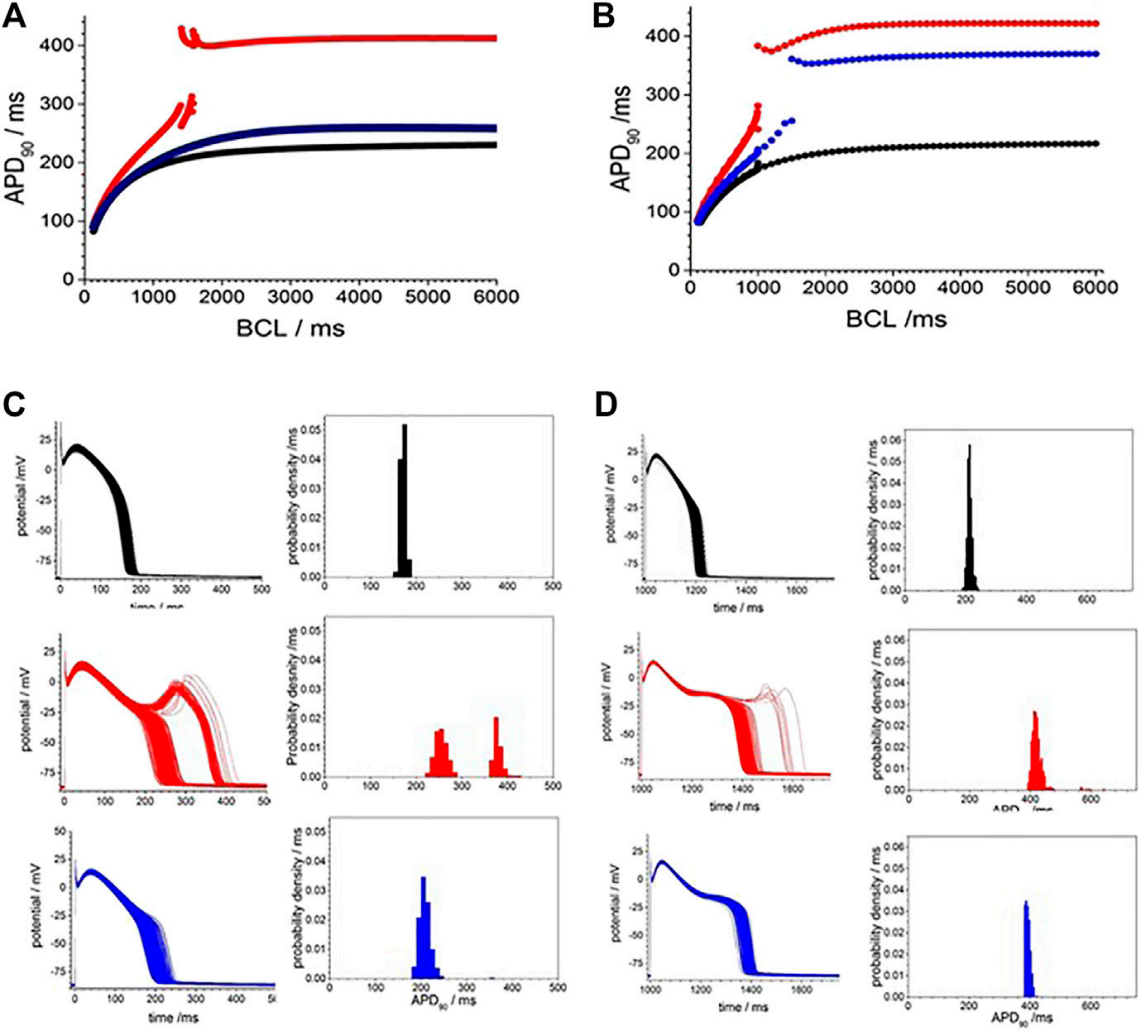
Action potential prolongation in Luo-Rudy dynamic model (LRd) by the effects of CO is reduced by the hERG modifier NS1643. Dynamic restitution curves for **(A)** endo- and **(B)** epi-cardial models with standard values (black). or modified for the effects of CO (red), or the effects of CO and NS1643 (blue). 100th periodic triggered action potentials, and their APD_90_ histograms, at BCL of 6s for population of **(C)** endo- and **(D)** epi-cardial cell models with all the current magnitude parameters Gaussian distributed with a 5% standard deviation, and the means of these parameters either their standard values (black), or modified for the effects of CO (red), or the effects of CO and NS1643 (blue).

The restitution curves of Figure 5A,B show effects of CO on endo-and epi-cardial LRd cell models. CO increased the APD_90_ at all BCL, the APD lengthens as BCL is increased, and at a BCL>∼1,000 ms, there is a jump in the APD produced by the addition of an EAD, and no multiple EADs are produced as BCL is further increased. For the endocardial cell model there is a widow of alternans between BCL of 1,410 and 1,560 ms. These CO effects are almost abolished in the endocardial cell model (Figure 5A) and reduced in the epicardial model (Figure 5) by simulating the addition of NS1643. In the epicardial model, the APD is reduced by the effect of NS1643, and the EADs begin occurring at longer BCL; 1,500 ms compared to 1000 ms. In the endocardial cell model the EADs are abolished, and the prolongation of the APD at a BCL of 6 s is reduced by 85%. For the population of cell models, with 5% variability in all the membrane maximal conductance parameters, the effect of CO on the endocardial models is to prolong the APD, and increase its variability, with EADS produced in <1% of the variant models. In the epicardial cell models the APD and its variability are increased producing EADs and a bimodal distribution of APD_90_. The EADs and the bimodality of the distribution are abolished by modelling the effects of NS1643, but the mean APD_90_, and its standard deviation are still larger than those of the population of standard cells.

### Carbon Monoxide Prolong Action Potential Duration in Human Induced Pluripotent Stem Cell Derived Cardiomyocytes

**FIGURE 6:**
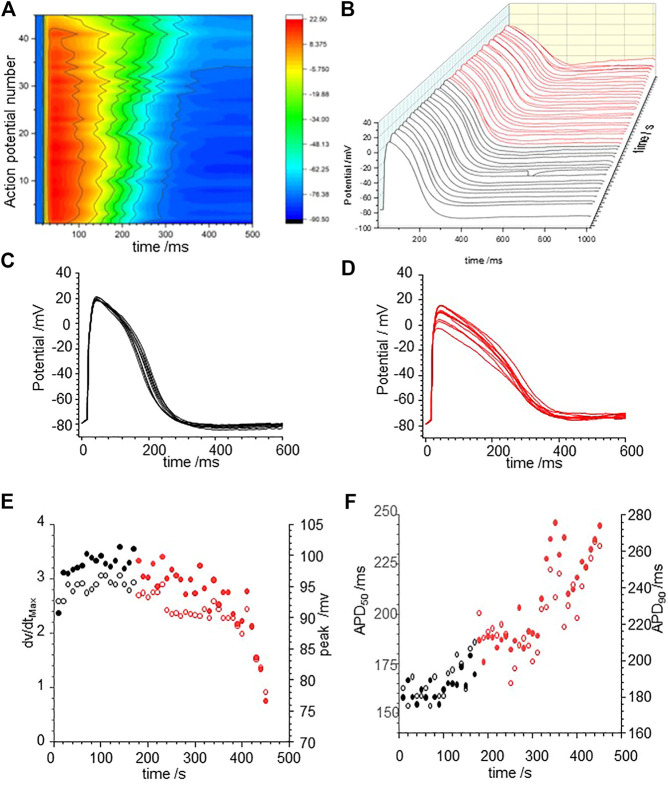
CO prolongs action potentials of human iPSC derived cardiomyocytes **(A)** Color map of membrane potential during 45 cycles of pacing, with BCL of 10 s **(B)** staggered plot of V(t) during these action potentials. Black before effect of CO, red during development of effect of CO. **(C)** Intrinsic fluctuations in APD_90_ before effect of CO. **(D)** Progressive lengthening of evoked actions potentials during CORM-2 prefusion. **(E)** CO effect on the maximum rate of depolarization and duration of AP. Time series plot of peak (filled symbols) and dV/dt_max_ (open symbols) measurements. CORM-2 was perfused into the bath supply line at time 180 s. APD_50_ (open symbols and APD_90_ (filled symbols) before (black symbols) and during (red symbols) the response to perfusion by CORM-2.

Human iPSC derived cardiomyocytes are genetically isomorphous with human ventricular cells, have action potentials that resemble those of developing foetal human ventricular myocytes, and have the same, but quantitatively different expression profile of membrane ionic channels (Mann et al., 2019; Kernik at al., 2019). Figure 6 illustrates the action potential responses to pacing with a BCL of 10 s of an isolated hiPSC cardiomyocyte at room temperature to CORM-2. In the initial 170 s, before CORM-2 application, the action potentials triggered by each stimulus are variable, with a stationary APD_90_ sequence with coefficient of variation of 5.6% (184.9 ± 10.3 ms). CORM-2 produced a gradual concurrent increase of APD_50_ and APD_90_ of the myocytes, by about 50% over 150 s/15 cycles; from 190 to 300 ms for APD_90_ in the example of Figure 6. The progressive lengthening of the APD_90_ was mirrored by a decrease in the dV/dt_max_ of the action potentials, from 2.9 ± 0.1 to 1.98 ± 0.49 Vs^−1^, and a decrease in the peak-to peak amplitude of the action potentials, from 97 mV to 76 mV. EADs were not observed in any of the paced cells (*n* = 4), either before or during exposure to CORM-2.

### Carbon Monoxide Effects Prolong Action Potential Duration in ORd Model

**FIGURE 7:**
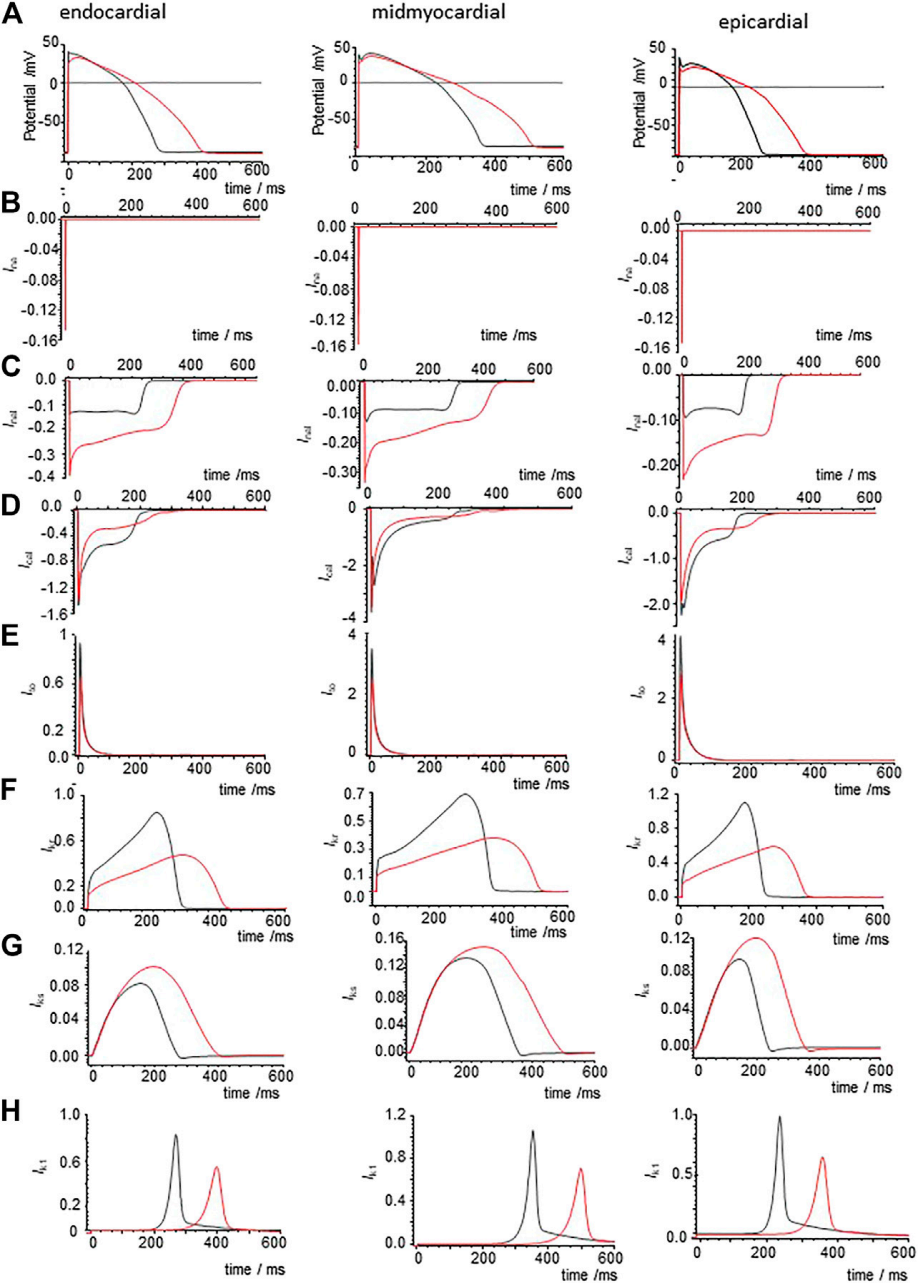
Ionic currents underlying action potential prolongation by the effects of CO on action potentials in the un-diseased ORd cell models. **(A)** Action potentials of endo-, midmyo- and epi-cardial cell models. 100th action potential at BCL of 1 s **(B)** I_Na_, **(C)** I_NaL_, **(D)** I_Ca,L_, **(E)** I_to_, **(F)** I_Kr_, **(G)** I_Ks_,**(H)** I_K1_ during these action potentials, all currents in μA/μF. Red: with CO parameters, black standard parameters.

Modelling the effects of CO by applying the percentage changes in maximal G_Na_, G_Na,l_, G_CaL_, G_Kr_, and G_K1_ from Table 2 to the standard O’Hara et al., 2011 models extends the APD of the three cell types at a BCL of 1 s (Figure 7A), with APD_90_ increasing by 51.4% (epicardial), 41% (midmyocardial) and 47% (endocardial). During pacing at 1/s the computed APs take ∼10 cycles to settle into periodicity, with no alternans or EADs observed. The maximal amplitude and shape of I_Na_ (t) are unaltered by the 53% reduction in G_Na_ (Figure 7B). In all three cell models the 105% increase in G_Na,l_ gives an approximate doubling the peak I_Na,L_ (increase of 40–80% during the upswing of the action potential), and during the plateau (increase of 25–42% at end of plateau, and the I_Na,L_ persists until the delayed rapid phase of repolarization begins. The decrease in P_CaL_ (Figure 7D) gives a 53–71% decrease in the plateau I_CaL,_ which also persists until the delayed rapid phase of repolarization. The decreases in G_kr_, and G_k1_ produce 44 and 34% decreases in maximal I_Kr_ and IK_1._ Peak I_Ks_ is increased by 10–23% (Figure 7F–H).

**FIGURE 8:**
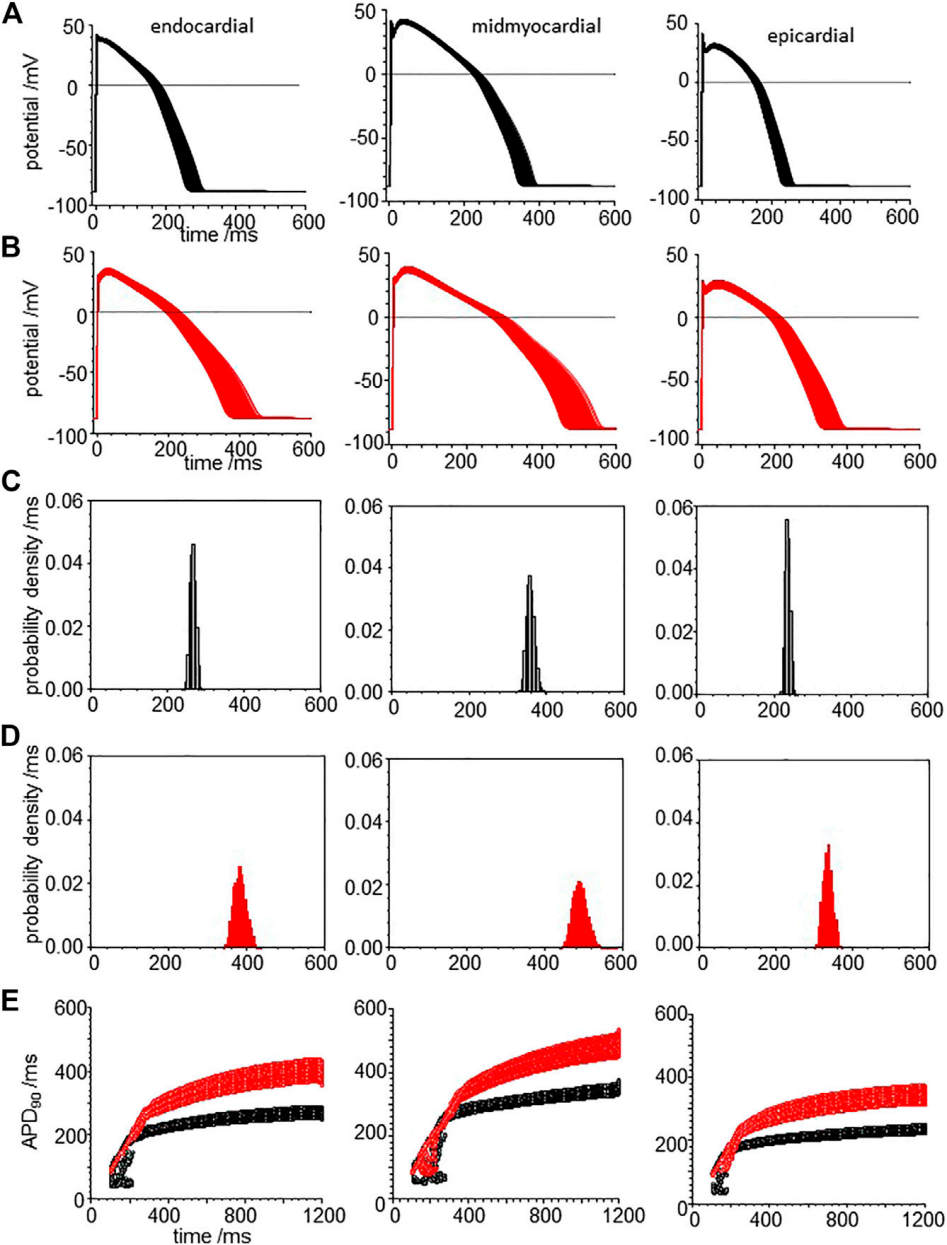
CO prolongs APD90 and increases its variability in ORd (2011) human ventricular endocardial, midmyocardial and epi-myocardial cell models. 1,000 action potentials at BCL of 1 S (100th action potential at BCL of 1 s) **(A)** for standard model **(B)** CO model with all the current magnitude parameters Gaussian distributed with a 5% standard deviation. Probability density estimates for APD_90_‘s of action potentials of **(C)** standard model and **(B)** CO model. **(E)** 32 superimposed dynamic restitution curves for cell models, with the conductance parameters distributed as above. Red: with CO parameters, black standard parameters.

When all the membrane current density parameters (G_Na_, G_NaL_, Gto. PCaL, GKr, GKs, G_K1_, G_NaCa_, G_NaK_), background currents (G_Nab_, G_Cab_.G_Kb_), sarcoplasmic Ca pump, and calcium handling parameters are Gaussian distributed with a 5% variability about their standard values, the AP of all three cell models are variable, but no EADs were observed (Figure 8A). The effects of CO on such a population of cell models is to increase the both the mean and standard deviation of APD_90_ (Figure 8, d and Table 3), and no EADs are observed at BCL from 300 to 1,200 ms (Figure 8E).

**TABLE 3:** CO increases variability: statistical moments of APD_90_ probability density estimates of Figure 8.

-	Endocardial	Midmyocardial	Epicardial	Endocardial with CO	Midmyocardial with CO	Epicardial with CO
Mean	266.1	358.5	235.2	383.6	490.9	335.7
% Coefficient of variation	2.9	2.9	2.8	4.09	3.7	3.6
Kurtosis	-0.41	-0.024	−0.37	−0.43	0.028	−0.43
skew	0.1	0.26	0.13	0.23	0.57	0.18

### Carbon Monoxide Effects Prolong Action Potential Duration and Induce Early After-Depolarizations in ORd Heart Failure Models

**FIGURE 9:**
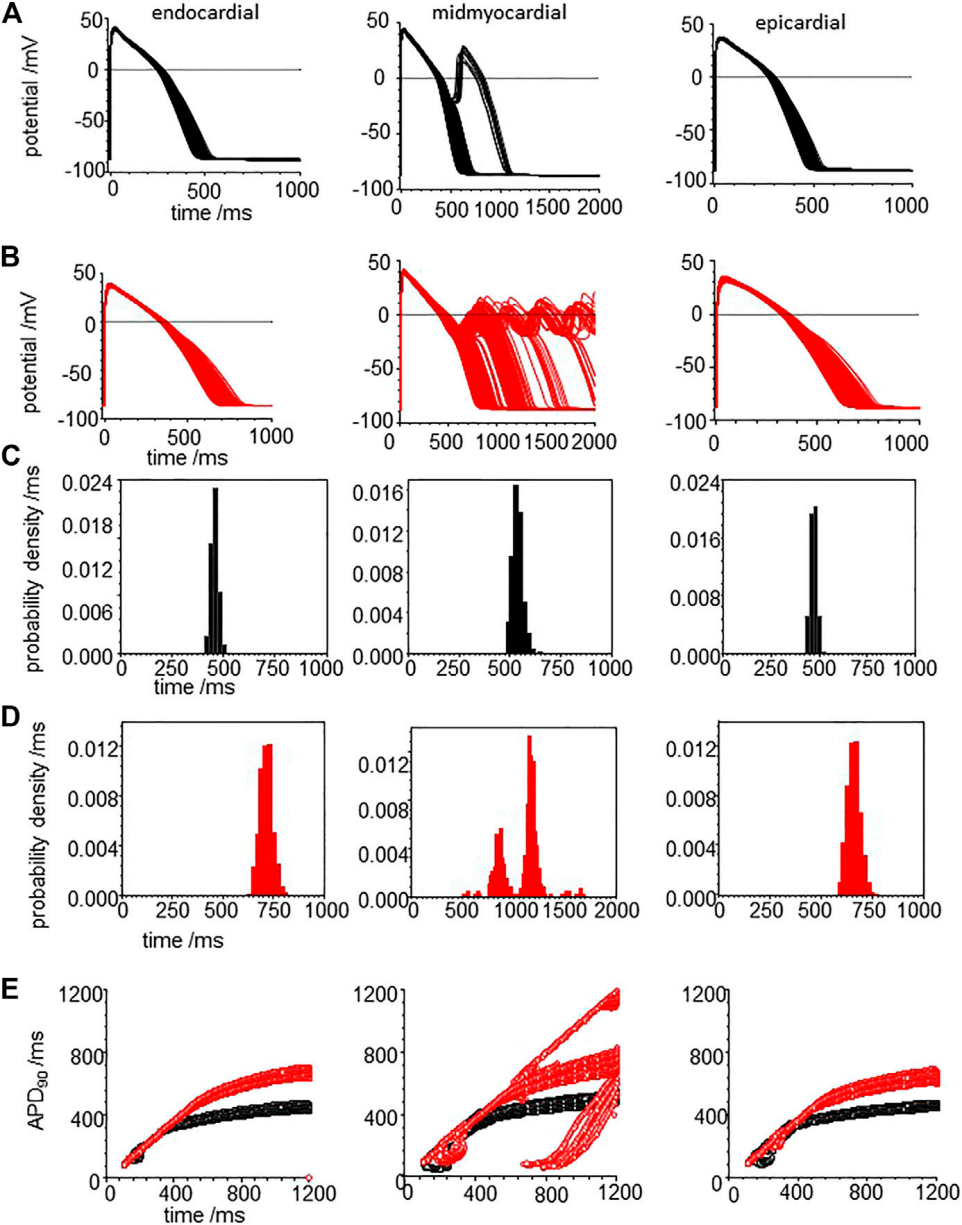
CO prolongs APD90 and increases its variability in the heart failure human ventricular myocardial cell models. Action potentials for **(A)** heart failure models, with means of the parameters from Elshrif et al. (2015) for heart failure and **(B)** for the effect of CO on the heart failure model. All the current magnitude parameters Gaussian distributed with a 5% standard deviation **(C,D)**. Probability density estimates for APD_90_‘s of action potentials of **(C)** standard model and **(D)** CO model. **(E)** 32 superimposed dynamic restitution curves for cell models, with the conductance parameters distributed as above. Red: with CO parameters, black standard parameters.

Tissue from failing hearts is more readily available than tissue from healthy hearts and so there is an extensive literature on the ionic currents and exchangers of myocytes from failing adult human hearts (Elshrif et al., 2015), and arrhythmogenic mechanisms in failing ventricles have been modelled (Ponnaluri et al., 2016). Heart failure is the end stage of a number of pathological processes, and the coefficient of variation of experimental measurements of the different maximal ionic currents in myocytes from heart failure patients ranges from <1 to 65%. Here we use the mean values from Elshrif et al., 2015 for the membrane current density parameters (G_Na_, G_NaL_, G_to_. P_CaL_, G_Kr_, G_Ks_, G_K1_, G_NaCa_, G_NaK_), background currents (G_Nab_, G_Cab_.G_Kb_), sarcoplasmic Ca pump, and calcium handling parameters, with a 5% variability about their standard values to produce heart failure myocyte model populations. APs, APD_90_’s and APD_90_ restitution curves are plotted for these heart failure model population in Figure 9.

Comparing Figure 9A–C with Figure 8A–C, the heart failure cell populations have ∼ two-fold longer APDs even without EADs, and <1% of the mid-myocardial cell models produced a solitary EAD. The effect of CO (Figure 9B–D) is to further lengthen the APD for endo-, midmyo-, and epicardial cell models (Supplementary Figure S3). No EADs are observed in the epi- and endocardial models, but single and multiple EADs are observed in the midmyocardial models. The midmyocardial cells were paced at a BCL of 2 s, to allow repolarization from multiple EADs, but 5% of the midmyocardial cell models fail to repolarize within 2 s. Estimates of the probability densities for APD_90_ of the endo- and epi-cardial populations of cells are unimodal and positively skewed, while the midmyocardial cell histograms are bi- and multi-modal.

The population restitution curves show bubbles of alternans at cycle lengths around 200 ms (Figure 9E), that CO has little effect on the APDs for cycle lengths < ∼400 ms, above which the APD of the CO-HF cell population with no EADs increasingly diverge from those of the HF population. In the midmyocardial CO model restitution curve up to a BCL of 1200 ms cells that produce EADS fail to repolarize within the cycle length.

### Carbon Monoxide Effects on the Vulnerability of Cardiac Tissue Models With ORd Cell Kinetics

Figure 8A–E illustrate the effects of CO produced changes in conductances and permeabilities on the electrical behaviour of single isolated, isopotential myocardial cells, cell models and populations of cell models. In myocardial tissue myocytes are electrically coupled, *via* gap junctions, to neighbouring myocytes and fibroblasts, and the depolarization of the action potential wavefront propagates through the tissue at a velocity of 0.1–0.5 m/s. The propagation velocity depends on wave properties - its curvature, and rate (the conduction velocity restitution relation, where conduction velocity decreases at short cycle lengths/high rates, as the wavefront is propagating into partially recovered tissue). It also depends on tissue properties: it is anisotropic, faster along the direction of the long axis of the myocardial cells, and changes as the wavefront spreads into tissue with a different impedance e.g. the boundaries of the myocardium, or anatomical structures. it depends on the cell-to-cell coupling conductances, and extracellular resistivity. It also depends on cell membrane properties; the maximal inward depolarizing current density, which drives both rising phase of the cell action potential, and the local circuit current that drives propagation, and the rate dependence of the action potential and ionic currents. The changes in the cell membrane conductance produced by CO will contribute to changes in propagating activity in myocardial tissue, and the membrane effects of CO on propagation can be computed using Eq. 1 with the CO produced conductances incorporated into the model for I_ion_(t). A constant diffusion coefficient *D* in Eq. 1 assumes that CO has no effect on tissue cell-to-cell coupling or intra:extracellar volume ratios.

**FIGURE 10:**
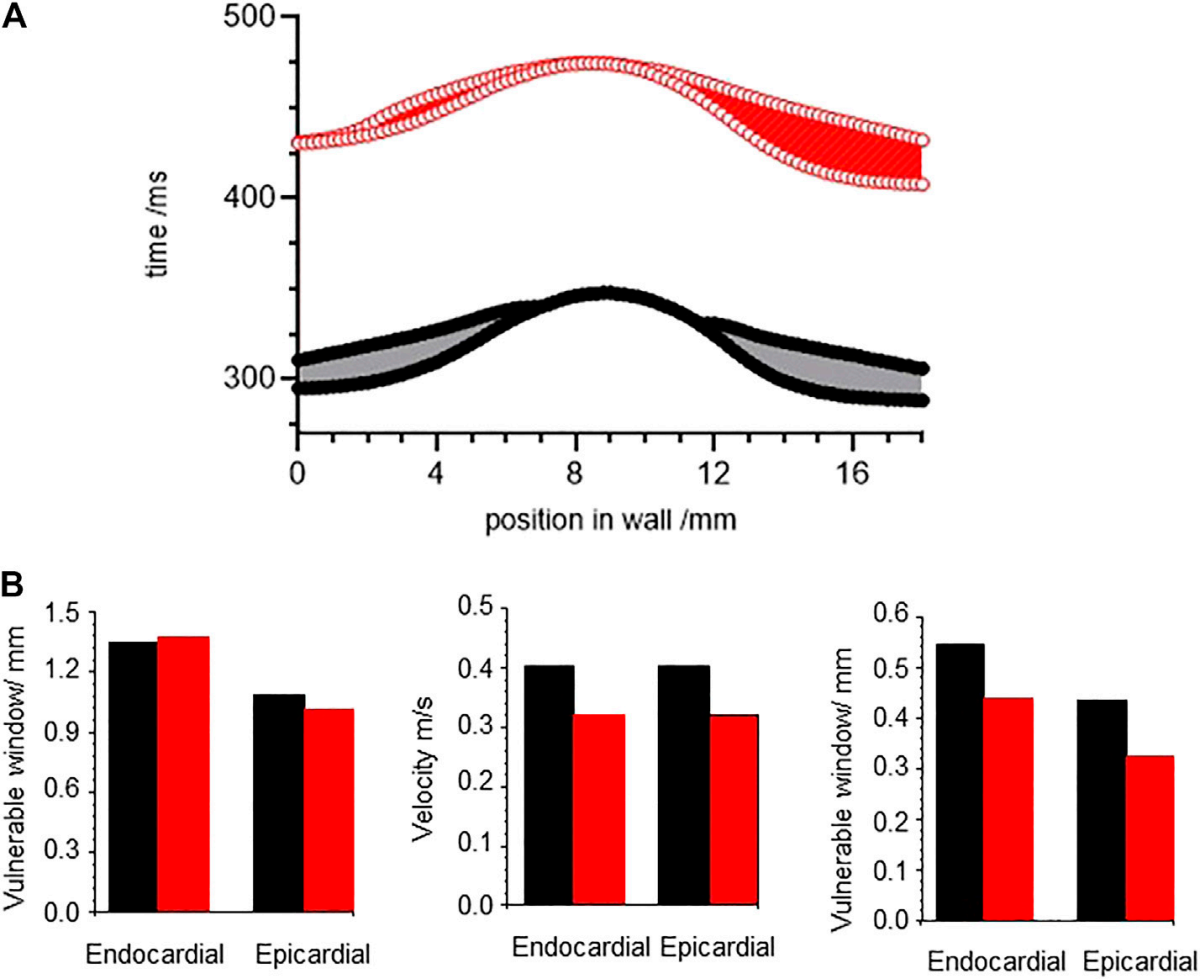
CO differentially changes vulnerable window. **(A)** temporal vulnerable window computed for an 18 mm one-dimensional ventricular wall ORd model, with equal thirds of endocardial, midmyocardial and epicardial tissue. **(B)** conduction velocity, wavelength, temporal and spatial vulnerable windows for one dimensional ORd models of homogeneous ventricular epicardial and endocardial tissues. Strand was paced at a BCL = 1000 ms to establish the initial resting conditions, and VW determined at each space step.

The temporal vulnerable window at a location in a tissue or point in a spatially extended tissue model is the narrow time interval following a propagating action potential during which a standard (1.5x threshold for resting tissue) stimulus produces unidirectional propagation: tissue in the direction of the preceding action potential has not yet recovered its excitability, while tissue in the opposite direction to the preceding action potential has recovered excitability. This is a time interval of unidirectional conduction block, which in a tissue can allow the initiation of re-entrant arrhythmic activity. Figure 10A, shows the temporal vulnerable window computed for an 18 mm one-dimensional heterogenous ventricular wall ORd model, with equal thirds of endocardial, midmyocardial and epicardial tissue, and tissue with standard parameters in black, and parameters for CO in red. Activity was initiated at the endocardial end of the strand, and conduction velocity (CV) computed through the central 12 mm of the wall for the initial action potential produced by the test stimulus. The start, end and width of the vulnerable window changes continuously through the strand model, as the diffusive coupling locally smooths changes in potential. The VWs are asymmetric, due to the endocardial to epicardial direction of propagation, differing endocardial and epicardial action potential durations, and boundary effects.

The temporal VW, conduction velocity and spatial VW - temporal VW X CV -were computed for one dimensional ORd models of homogeneous ventricular epicardial and endocardial tissues (Figures 10B–D). The effects of CO change the temporal VW by < 5%, decrease the velocity by ∼20%, resulting in a decrease in the spatial vulnerable window of 20–25%.

## Discussion

The experimental data presented in Table 2 give changes in maximal ionic currents following exposure to CO via CORM-2. The maximal ionic currents for a specific channel type depend on the density of membrane channels, the cell surface membrane area, both of which vary between cells from the same tissue, and so are given as % changes from their control values. We use these percentage changes to scale the maximal conductance parameters of the cell models used. This neglects any effects CO might have on the channel kinetics, but these would be partially incorporated into the experimental maximum and peak current measurements. There are different computational models for action potentials mammalian ventricular myocytes, and for the same myocyte type in the same species. New models, that either add to existing models by incorporating new experimental results, or re-interpret existing results, are continually being developed. There is a general consensus of the membrane dynamics, with differences in the number, roles, and detailed kinetic of the ion-specific currents that comprise the membrane ionic current, but less consensus about the intracellular ionic fluxes, and their spatial aspects. For any species specific, individual myocardial cell type there can be a number of published computational models for their action potentials, all of which are of the form dV/dt = −I_ion_, which differ in their parameters, variables and the formulation for their voltage dependent kinetics and intracellular ion binding, transport and sequestration. The models used were selected as they are new enough to be based on cell and single channel patch clamp data, and old enough for them to been validated by their use in reproducing or explaining experimental data from isolated cell or tissue preparations, and interpreting *in vivo* recordings. They have also been used in computational studies of pathologies (e.g channelopathies (Wu et al., 2019)), where *in vitro* experiments are not practical, and in the target design and development of novel putative pharmaceuticals (Trayanova et al., 2012). They are convenient as their code is in the public domain and run on standard laptops. They are fit for the purpose of reproducing V(t) recordings from single cells, predicting V(t) activity in single cells and tissue in pharmacological or modified pathological conditions, and in interpreting the ionic current and dynamical mechanisms that produce different behaviours. The modelling of concurrent recordings of V(t) and intracellular [Ca^++^](t) or spatially varying or stochastically fluctuating [Ca^++^] would require sarcomere-level models with more detailed intracellular ion transport, binding, and sequestration.

The three models we used are; the Gattoni et al., 2016 (rat) and Luo and Rudy, 1994 (guinea-pig) and O’Hara et al., 2011 human models were selected as being species appropriate, convenient, and fit for purpose. They, and their modifications, have been widely used so there is an extensive literature for comparison (Viswanatahan and Rudy, 1999; Heijman at al., 2011; Cardona et al., 2016; Gattoni at al., 2017; Lee at al., 2017; Whittaker at al., 2017). Here we use the ORd (2011) model to predict pro- or anti-arrhythmogenic effects in adult human tissue, as CO poses issues in public health and clinical medicine and human *in vitro* and *in vivo* electrophysiological data is lacking. There are models for hiPSC derived myocytes (Kernik et al., 2019) but the hiPSC derived myocytes tend to have an “immature”, more depolarised resting membrane potential of −40 to −70 mV, too low for activation of I_Na_ (Goversen et al., 2018), which can be compensated by injection of hyperpolarising current. hiPSC models are useful for investigating membrane processes, such as pharmacological actions on ion channels in human myocytes, and can be used for investigating cell processes, such as effects on repolarization and APD. They are not suitable for the investigation of arrhythmogenic mechanisms, which is a tissue level phenomenon influenced by propagation velocity, which is influenced by maximal inward I_Na_ and cell-cell coupling. Fabrication of tissue from hiPSC derived myocytes (Beauchamp et al., 2020) could provide the propagation velcity data for using hiPSC models in tissue computations.

The experimental results of Figures 1, 3, 6 all show CO induced effects on depolarization processes; a decrease in dV/dt_max_ and peak amplitude of the action potential; and repolarization processes; an increase in the APD, due to slowing of the rate of repolarization, and changes in the stability of the plateau, evidenced by its extension and oscillations/multiple EADs. The changes in the ionic currents are quantitative and can produce qualitative changes in the shape of the evoked action potentials.

### Carbon Monoxide Produced Changes in Depolarization Processes

The decreases in dV/dt _max_ and peak of the action potential are to be expected from the modeled 53% reduction in G_Na,_ and are quantitatively mirrored by the computed reductions in the three models. The control values of dV/dt_max_ are higher in the three computational models than in the experiments. A decrease in G_Na,_ is the basis for class 1 antiarrhythmic agents, and in tissue will produce a decrease in conduction velocity, and so can suppress tachycardia. It can also be proarrhythmogenic, as demonstrated in the cardiac arrhythmia suppression trial (Akhtar et al., 1990).

### Carbon Monoxide Produced Slowing and Delayed Repolarization

Figures 1B, 6D and from 200 to 400 s in Figure 3A,B,D all show a gradual prolongation of the action potential by slowing the rates of repolarization: this is explained by an increase in slow depolarizing currents (I_NaL_), or a decrease in repolarizing currents (I_Kr_. I_K1_) resulting from the CO induced increase in G_NaL_ and decreases in G_Kr_. G_K1_. The Gattoni et al. model does not incorporate a G_NaL_, and results in Figure 2 do not include an I_NaL._ Incorporation of the Hund and Rudy (2004) formulation for canine I_NaL_ into the model, with the same ration for G_Na_:G_NaL_(not shown) does produce further prolongation of the action potential and lead into EADs. The profile of repolarizing ionic currents (I_Kr_. I_Ks_, I_K1_) differ between the endocardial and epicardial LRd and ORd cell models, but all three cell models can produce qualitatively similar behaviors. The hERG activator NS1643 reverses the CO induced prolongation in guinea pig cells (Figure 3) and the LRd models (Figure 4), consistent with the major role of I_Kr_ in repolarization in the guinea pig ventricular myocyte.

### Carbon Monoxide Produced Changes in Action Potential Duration

The effects of CO on action potential shape, prolongation, EADs, extended plateau with oscillations are qualitatively reproduced by the modeled effects of CO but the model APD are less than the *in vitro* APDs.The cell models are all constructed to represent behavior at body temperature of ∼37**°**C, and one would expect the models to be faster and the model APDs to be shorter than seen in the room temperature experiments. In guinea pig papillary tissue cooling by 10**°**C prolongs APD at all cycle lengths and increases the steepness of the restitution curve (Bjørnstad et al., 1993). Temperature enters into the cell models explicitly (*via* the Nernst equilibrium potential equation, and implicitly *via* the temperature dependence of the rate coefficients of the membrane current variables, and the intracellular binding and sequestration processes. The temperature dependence can be quantified in experiments by the Q_10_, and data is available for many mammalian membrane ionic conductance kinetic processes, but data is not available for all the intracellular binding and sequestration processes, where processes limited by diffusion will have Q_10_ ∼1 and processes relating to conformational changes in proteins closer to 3. Since the different processes have different Q_10_ the shape as well as the duration of the action potential will change.

### Carbon Monoxide Produced Changes in Plateau Stability and Early After-Depolarizations

**FIGURE 11:**
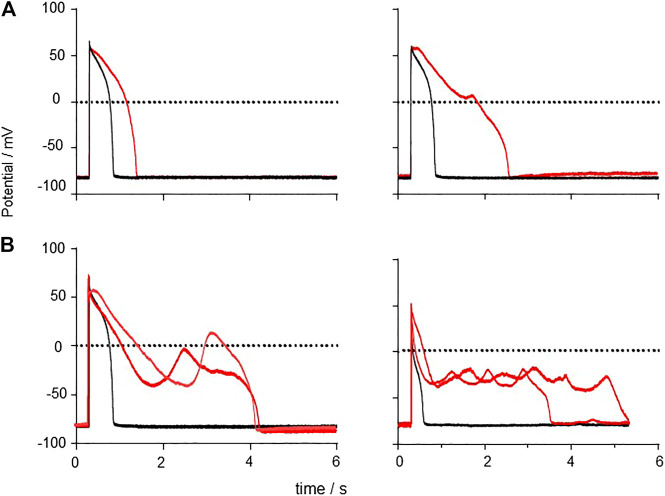
Generation of EADs by CO Examples of responses to CO, APs and EADs from four different cells. **(A)** action potential prolongation **(B)** initiation of a failed EAD around V = 0 mV **(C)** initiation of EAD from v∼50 mV **(D)** multiple EADs/oscillations.

The effects of CO on guinea pig myocyte action potentials are illustrated in Figure 11, by selected action potentials from four different preparations. The prolonged responses are after different exposure times to CO, with longer responses from longer exposures-see Supplementary Figure S1. There is no temporal evolution from a to b to c to d. The panels illustrate different types of response; 1) simple prolongation of the action potential, by a decrease in the rate of repolarization; 2) repolarization is interrupted when the membrane potential is still positive by an abortive EAD, that fails to develop; 3) the membrane potentials repolarizes to ∼ −45 mV when a full EAD develops; 4) the plateau is extended by multiple EADs, or by oscillations around the plateau. These different behaviors may be described by considering the stability of the plateau membrane potential, and how it is changed by changes in the membrane conductance parameters. If it is stable, the plateau will persist, as in the cells that fail to repolarize. If it is weakly unstable, the membrane potential will show oscillations that increase in time, as Figure 11D, where the amplitude of the oscillations grows in time until they trigger a fast repolarization. This description may be formalized in terms of bifurcation theory, that describes how qualitative changes in behavior develop in dynamical systems modeled by differential equations and has been applied to simple and biophysically detailed models of cardiac EADs (Tran et al., 2009; Kügler, 2016; Kurata et al., 2020). In this approach, different qualitative behaviors are associated with different compact regions of parameter space, and changes in behavior (bifurcations, say from a stable equilibrium to an unstable equilibrium, as stable oscillations emerge) are associated with movement in parameter space across the bifurcation boundary separating these regions.

### Variability

Any individual cell model can be partially characterized by a set of parameters, for example, the principal membrane current parameters (G_Na_, G_NaL_, G_to_. P_CaL_, G_Kr_, G_Ks_, G_K1_, G_NaCa_, G_NaK_), of the ORd model, and with each parameter considered as an axis in a high dimensional parameter space, the particular set of parameter values defines a point in this space. The effect of CO is to change some of these parameter values, to a different point, which is associated with a quantitatively different solution, or action potential. This quantitatively different action potential solution may be qualitatively similar (such as a lengthened action potential) or qualitatively different (such as a response with an oscillatory plateau), in which case the two points are separated in parameter space by a bifurcation. In the cell population models, the collection of models defines a cloud of points in parameter space, and for Figures 2C,D both clouds overlaps the bifurcation into an EAD, and in Figures 5C,D the standard and +CO + NS1643 clouds are on the action potential side of the bifurcation, and the +CO cloud overlaps the bifurcation, and in Figure 8A,B the clouds are on the action potential side of the bifurcation. This redescription changes the computational problem from one of multiple Monte Carlo simulations of the cell models with randomly selected parameters sets to a deterministic problem-find the bifurcation “hypersurface” in parameter space that separates action potentials from action potentials with EADs. This approach can be applied to any pharmacological agent whose actions on parameters in the cell electrophysiology models have been quantified.

In the population cell models the membrane current parameters are selected from a Gaussian distribution about a mean equal to the deterministic value, with a standard deviation of 5%., and for each parameter outliers more than three standard deviations away from the mean are excluded i. e ∼99.7% are included. There are 14–16 parameters in the cells models that are varied concurrently, and so large numbers of simulations would be required to adequately sample the parameter space, Experimental measurements of ionic conductances are based on 4–14 cells, in the heart there are 10^9^ myocytes, and so for the illustrative computations 10^4^ was selected as both practical and reasonable. The APD_90_ histograms are positively skewed, with more longer APDs than a Gaussian distribution about the same mean, with the same standard deviation. The effect of CO is to increase the variability in the APD_90_’s, in particular, it increases the very low likelihood of a long APDs in all the cell model types.

### Pro- and Anti-Arrhythmogenicity

Cardiac arrhythmia are changes from the pattern of propagation during normal sinus rhythm, with near synchronous excitation of the atria, followed by near synchronous excitation of the ventricles, to abnormal, perhaps re-entrant and irregular, propagation. Although sudden cardiac death accounts for 15–20% of all deaths, individual arrhythmia are rare, unpredictable events. For an arrhythmia to have a clinical impact it needs to be initiated, and to persist and not self-terminate within a few seconds. The initiation and persistence of an arrhythmia are separate processes, with initiation produced by an ectopic source, or by a wave break. These are both tissues, not cell level phenomena. The increase in isolated cell APD produced by CO, seen in experiments and models, is assumed to be pro-arrhythmogenic, as long QT syndromes, in which there is an increased risk of re-entrant arrhythmia and sudden cardiac death, are associated with prolonged ventricular APDs. The production of prolonged plateau and multiple EADs by CO seems to be self-evidently proarrhythmogenic. However, these changes in an individual cell will be electrotonically smoothed by intercellular coupling, and insufficient to initiate an ectopic propagation, or produce a wave break, unless they occur synchronously in a compact cluster of 10^4^ cell. Increases in the differences between APD in different regions of tissue (dispersion of APDs) will accentuate any normal or pathological heterogeneities. Such dispersion of APD is increased in the cell populations simulations, and the increase in variability, especially in extending the extremes of the APD probability distributions, rather than the increase in deterministic or mean APD, is a stronger driver of risk of arrhythmia. This increase in variability is increased when the cloud of cell population parameter overlaps a bifurcation: both APD prolongation and EADs act via increasing the increased variability increasing the APD dispersion. The computations illustrated are for the effects on standard models for healthy tissue; the increased variability is exaggerated in models with parameters based on experiments from heart failure. An increase in APD variability and dispersion amplify electrophysiological heterogeneities and alter the trigger for an arrhythmia, by allowing current from depolarized tissue to recovered tissue. The effectiveness of localized currents to initiate unidirectional propagation, and hence re-entry, is quantified by the vulnerable window. The CO produced changes in temporal vulnerable window are overwhelmed by the CO produced changes in conduction velocity, giving a reduced spatial vulnerable window, or volume of tissue where unidirectional propagation can be initiated.

## Conclusion

We have reproduced the CO effects on action potential morphology seen *in vitro* in rat and guinea pig ventricular myocytes; action potential prolongation, early afterdepolarizations (EADs) and plateau oscillations. The computations show the effects over the physiological and experimental range of rates. The modeled effects of CO increases the variability in APD produced by variability in membrane conductance parameters, and so predicts cell population responses. In human cell and tissue models the effects on action potential dispersion, variability and heterogeneity, which lead to the current for initiating an arrhythmia, are proarrhythmogenic. These proarrhythmogenic effects of CO are more pronounced in the heart failure cell models than in the healthy cell models. The effects on the spatial vulnerable window, the amount of tissue within which a suprathreshold current can initiate unidirectional propagation, is reduced, and so is anti-arrhythmogenic.

## Data Availability

The original contributions presented in the study are included in the article/Supplementary Material, further inquiries can be directed to the corresponding author.
