# Supplementary material for: Deterministic and Stochastic Cellular Mechanisms Contributing to Carbon Monoxide Induced Ventricular Arrhythmias
Source: Front Pharmacol. 2021 Apr 28;12:651050. doi: 10.3389/fphar.2021.651050 (PMC8113948; doi:10.3389/fphar.2021.651050)
Supplement: Supplementary file 1 [file presentation1.pdf]

Supplementary Material.

Deterministic and Stochastic Cellular Mechanisms Contributing to Carbon Monoxide Induced Ventricular Arrhythmias

Moza M. Al-Owais[1], Derek S. Steele[1], Arun V. Holden[1], Alan P. Benson[1]

[1] School of Biomedical Sciences, University of Leeds, Leeds LS2 9JT, UK

*** Correspondence:**
M. M. Al-Owais
m.al-owais@leeds.ac.uk

Supplementary Fig S1

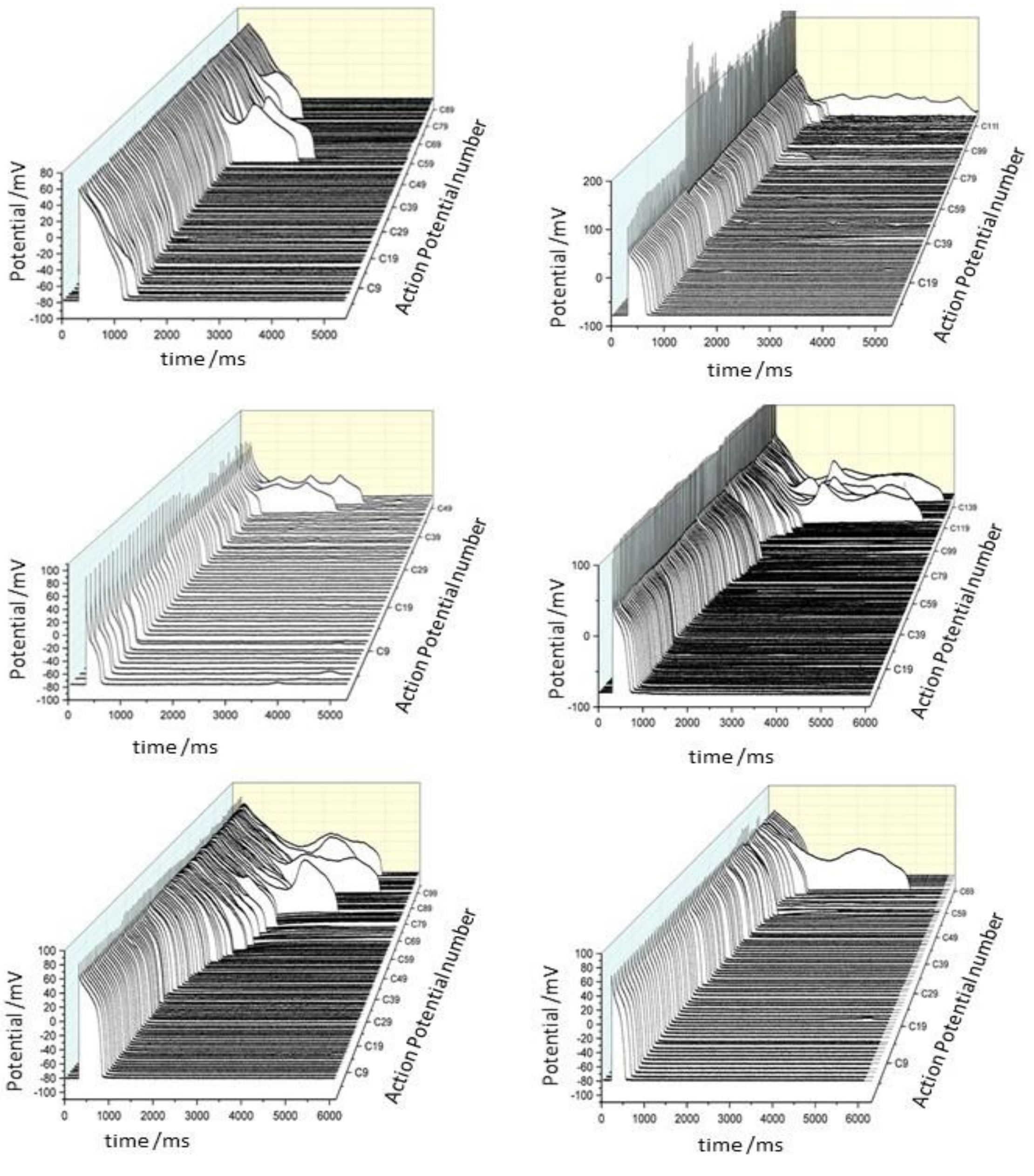

Figure S1. Stability of cell recordings, time course of development of response to CORM-2, and intermittent EADs. CO prolongs action potentials of isolated guinea-pig ventricular myocyte**.** Each panel displays action potentials of a guinea pig single myocyte that is periodically stimulated with a BCL of 6 s and plots consecutive action potentials before and during perfusion with CORM-2, as in Figure 3b. The action potentials before application of CORM-2 are stable, and exhibit fluctuations in APD. Perfusion with CORM-2 leads to a gradual, progressive increase in APD. The smooth increase in APD is irregularly interrupted by EADs and multiple EADs.

Supplementary Fig S2

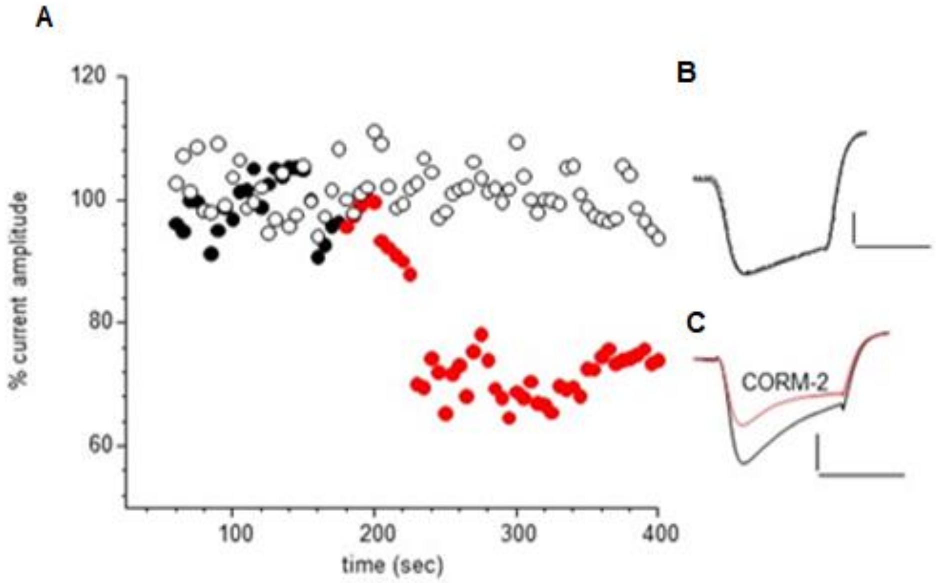

Figure S2. Stability of conductance changes during repetitive clamping. CORM-2 inhibition of L-type Ca^{2+} currents in rat cardiomyocytes. Currents were evoked by a step depolarization from -30 to +10 mV for 75 ms and a cycle length of 5s.(a) time series examples of % of peak amplitude showing either minimum run down in control (no CORM-2; black open symbols) or before (black filled symbols) and during (red filled symbols) the prefusion of CORM-2 (3µM). Sample traces from (a) are shown in b and c, each is the average of 10 sequential traces taken immediately before 180 s (black line, b and c) and after 350 s dashed black line (b) and red line (c), Scale bars, 200pA (vertical) and 20ms(horizontal) in each case.

Supplementary Figure S3

	Standard CO APD			Heart Failure CO APD		
	mean	sd	cv	mean	sd	cv
endocardial	267.3	7.9	0.04	386.4	15.9	0.04
midmyocardial	350.1	8.04	0.02	484.5	17.4	0.03
epicardial	233.7	6.3	0.02	336.5	12.5	0.03

Supplementary Table 1. CO effect on APD in standard cells and heart failure models.
